# Phosphorylation of the protamine-like protein from baculovirus affects its DNA binding and virus propagation

**DOI:** 10.1093/nar/gkaf506

**Published:** 2025-06-12

**Authors:** Qian Cheng, Siqi Zhu, Yanrong Lv, JiongJiong Liu, Qinglin Su, Meijin Yuan, Yang Liu, Kai Yang

**Affiliations:** State Key Laboratory of Biocontrol, Sun Yat-sen University, Guangzhou 510275, China; School of Biomedical Sciences, The University of Hong Kong, Hong Kong SAR 999077, China; State Key Laboratory of Biocontrol, Sun Yat-sen University, Guangzhou 510275, China; State Key Laboratory of Biocontrol, Sun Yat-sen University, Guangzhou 510275, China; State Key Laboratory of Biocontrol, Sun Yat-sen University, Guangzhou 510275, China; State Key Laboratory of Biocontrol, Sun Yat-sen University, Guangzhou 510275, China; School of Biomedical Sciences, The University of Hong Kong, Hong Kong SAR 999077, China; State Key Laboratory of Biocontrol, Sun Yat-sen University, Guangzhou 510275, China

## Abstract

Viral DNA-binding proteins, a type of protamine-like protein, pack the viral genome and promote late viral gene expression. In baculovirus *Autographa californica* multiple nucleopolyhedrovirus, DNA-binding protein P6.9 plays a crucial role in the production of progeny viruses. Earlier studies suggested the importance of P6.9 phosphorylation states in its function, while the detailed mechanism remains elusive. Here, we demonstrated that permanent hypo- and hyper-phosphorylation on the N-terminus of viral P6.9 significantly inhibits the proliferation of progeny viruses. Our data revealed that phosphorylation changes the DNA binding affinity of P6.9, which determines the distinct localization of P6.9 in the host nucleus. Both viral and host gene expressions were largely up or downregulated by the phosphorylation states of P6.9, indicating the central role of P6.9 in governing the tempo of viral replication. Our work provides an intriguing model of how the phosphorylation/dephosphorylation dynamics of P6.9 regulate viral packaging and gene expression patterns, which sheds light on the general principle of viral–host interaction.

## Graphical Abstract

**Graphical Abstract:**
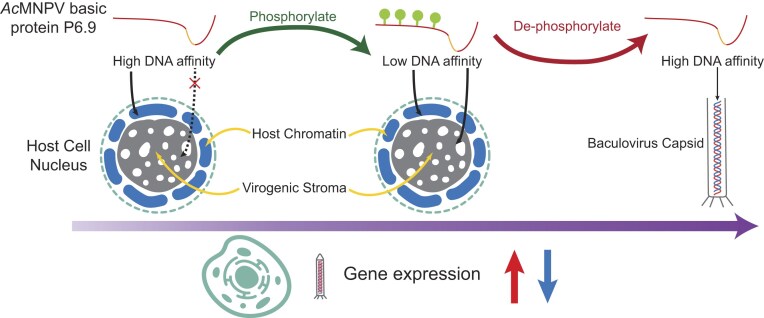

## Introduction

Eukaryotic genomes are efficiently condensed and packaged into chromatin by histone proteins to preserve genetic information. However, during spermatogenesis this function of somatic histone is supplanted by other small basic proteins called sperm nuclear basic proteins (SNBPs), which can be grouped into three categories: protamine (P-type), protamine-like proteins (PL-type), and histone H1-type proteins (H-type). The transition from histones to SNBPs ensures effective DNA protection and faithful transmission of genetic information [1–5]. However, most viruses do not possess the histones/SNBPs but instead utilize unique DNA-binding proteins to play a crucial role in the viral packaging process. For instance, the DNA packaging of herpes simplex virus requires the coordinated involvement of seven viral proteins, including the nonspecific DNA-binding protein UL15, which participates in the translocation of DNA into the capsid with other proteins [6, 7]. Adenovirus core proteins V, VII, and μ are analogous to histones, possessing a high proportion of basic amino acids that facilitate nonspecific binding to DNA and its subsequent condensation [8, 9]. Notably, baculoviruses, widely studied and utilized DNA viruses, possess a small and arginine-rich basic protein commonly referred to as P6.9. P6.9 is highly conserved among baculoviruses to condense the viral genome, thereby functioning as a protamine-like protein [10–14].

Protein phosphorylation is the most abundant and highly significant post-translational modification (PTM) [15, 16]. As a reversible and rapidly responsive PTM, phosphorylation is involved in regulating protein interactions, conformation, and localization, which modulate a wide range of cellular biological processes. The addition and removal of phosphate groups typically occur on serine, threonine, and tyrosine residues. In living organisms, this process is involved in maintaining homeostasis, thereby regulating metabolism to adapt to changes in the cellular environment [17].

Proteomic characterization of *Autographa californica* multiple nucleopolyhedrovirus (AcMNPV), the baculovirus type species, has revealed dynamic coexistence of non-phosphorylated and scores of phosphorylated P6.9 forms during infection, with site-specific mapping identifying 13 distinct serine/threonine modification sites [18]. As viral DNA replication progresses, the host chromatin becomes progressively marginalized and a high-electron-density chromatin-like structure emerges at the center of the nucleus. This structure, known as virogenic stroma [19], serves as the site for viral DNA replication and capsid assembly [20]. During the advanced phase of viral infection, P6.9 expression exhibits progressive upregulation concomitant with the appearance of heterogeneous phosphorylation species characterized by multi-level phosphorylation states. Later on, the phosphorylation level of phosphorylated P6.9 first continuously increases and then gradually decreases until it almost disappears [21]. Meanwhile, a significant amount of P6.9 co-localizes with host chromatin at the inner nuclear membrane. Immunoelectron electron microscopy has revealed that the total natively phosphorylated P6.9 co-localizes with newly synthesized viral DNA within the VS, while non-phosphorylated P6.9 primarily locates at the edge of the stromal mattes of the VS and on the solid nucleocapsids within the VS [21]. These observations provide preliminary evidence for a dynamic phosphorylation–dephosphorylation cycling of P6.9 during its nuclear translocation (VS targeting) and subsequent viral genome encapsidation. However, the functional coordination among these post-translational modifications, their spatiotemporal regulation, and the resulting biological outcomes remain to be fully elucidated. In this paper, we primarily focus on the impact of serine/threonine phosphorylation of P6.9 on its DNA binding affinity and functional activity. We found that the dynamics of this process at the P6.9 N-terminal region is crucial throughout the entire viral infection cycle. We also proposed a model in which the virus regulates the phosphorylation level of P6.9’s DNA-binding domain to control its localization and functional dynamics within the nucleus.

## Materials and methods

### Cells and antibodies

*Sf9* (*Spodoptera frugiperda* IPLB-Sf21-AE clonal isolate 9) insect cells were grown in monolayer cultures at 27°C in Grace’s medium (Invitrogen Life Technologies) supplemented with 10% heat-inactivated fetal bovine serum, penicillin (100 μg/ml), and streptomycin (30 μg/ml). Rabbit monoclonal anti-hemagglutinin (HA) antibody was purchased from Abcam. Mouse monoclonal anti-H4 antibody was purchased from Abmart.

### Construction of plasmids and viruses (Fig. 2A)

The bP6.9KO (P6.9-deficient viral expression vector) was constructed by replacing a 48-bp fragment of *p6*.9 in bMON14272 with a CmR gene via Red/ET homologous recombination [21]. The *p6*.9-rescued virus (vP6.9-HA) was constructed by transposing the *p6*.9 tagged with an HA epitope (including the p6.9 promoter sequence and SV40 polyadenylation signal) as well as the *polh* and *mcherry* genes into the bP6.9KO. Furthermore, different combinations of serine/threonine phosphorylation sites in P6.9 are mutated to alanine or glutamic acid to construct vP6.9M-HA. The pUC57-P6.9M-HA plasmid, which contains the mutant P6.9 sequence fused with an HA tag at the C-terminus, was synthesized by Guangzhou IGEE Biotechnology Co., Ltd. SacI and BamHI restriction enzyme recognition sites were introduced at both ends of the plasmid. Subsequently, P6.9-HA and P6.9M-HA were transferred into the plasmid pUC18-PP6.9-SV40 (containing the p6.9 promoter sequence and SV40 polyadenylation signal) using double enzyme digestion and ligation [18]. This connection involved the native promoter and SV40 polyadenylation signal. Next, the fragment was inserted into the transposon vector pFastBac-PC (containing the *polh* and *mcherry* genes) using the EcoRI and XbaI restriction enzyme recognition sites on the vector. Finally, the complemented recombinant baculovirus and mutant recombinant baculovirus were constructed by transposing it into bP6.9KO as described previously [22].

### Virus growth curve

*Sf9* cells (1.0 × 10^6^ cells/35-mm-diameter dish) were transfected with vP6.9-HA or mutant recombinant viruses in triplicate with 1 μg bacmid DNA. The BV-enriched supernatant was then harvested at 24, 48, 72, 96, and 120 h post-transfection (p.t.). The virus titer was determined in Sf9 cells using a TCID_50_ endpoint dilution assay and calculated by the Reed–Muench method as described elsewhere [23–25].

### Plasmid construction for protein expression and purification

The MBP tag was amplified and fused at the N-terminus of P6.9. The FLAG tag was amplified and fused at the C-terminus of P6.9. Fragments of P6.9 were amplified by primers 6.9-F (GCAGACTGGAGGAATGGTTTATCGTCGCCGT) and 6.9-R (CGCAATAGTAGCGTGTTCTGTAACTTCGG). All PCR fragments were purified and cloned into pET16b-His vector to get the construct of pET16b-His-MBP-P6.9-FLAG. Positive clones were verified by sequencing.

Similarly, P6.9 mutants NA (7A) and NE (7E) were amplified using primers 7A-F (AGCAGCAGGTGCAGCATATGGTGCGGCACGCAGGCGCAGAAGC), 7A-R (GCTGCACCTGCTGCTGCACGGCGACGGCGACGATAAAC), 7E-F (AGAGGGTGAGGAATATGGTGAGGAACGCAGGCGCAGAAGCT), and 7E-R (TATTCCTCACCCTCTTCTTCACGGCGACGGCGACGATAAAC), respectively. All PCR fragments were then purified and cloned into pET16b-His vector to get the construct of pET16b-His-MBP-P6.9NA-FLAG and pET16b-His-MBP-P6.9NE-FLAG. Positive clones were verified by sequencing.

### Protein purification

P6.9 and mutants (NA and NE) were expressed in the *Escherichia coli* strain BL21-CodonPlus. The cells were grown in YT medium at 37°C until OD_600_ of 0.5–0.6, and IPTG (final concentration at 1 mM) was added to induce the protein expression at 16°C for 20 h before harvesting the cells.

The cell pellet was resuspended in lysis buffer containing 50 mM Tris (pH 7.4), 500 mM NaCl, 0.01% sarcosyl, 5% glucose, 1 mM PMSF, 0.5 mg/ml lysozyme, protease inhibitor cocktail mini-tablet (EDTA-free) (MCE), DNaseI (NEB), 0.5 mM CaCl_2_, and 2.5 mM MgCl_2_. After sonication (on 1 s, off 1 s, total on 30 s, three rounds), the cell lysis was centrifuged for 45 min at 18 000 rpm. The insoluble sediment was washed with buffer three times (50 mM Tris, pH 7.4, 500 mM NaCl, 0.01% sarcosyl, 5% glucose, 1.5% Triton X-100), and only the last time Triton X-100 was removed. The insoluble sediment was denatured and sonicated with 6 M Gu-HCl, 50 mM Tris (pH 7.4), and 30 mM imidazole. The soluble fraction was collected by centrifugation, and the supernatant was incubated with nickel agarose beads (high density) (GoldBio) for 1.5 h at room temperature (RT) . The beads were then incubated with buffer (2 M NaCl, 8 M urea, 50 mM Tris, pH 7.4, 30 mM imidazole, 0.01% sarcosyl, 5% glucose) for 40 min, and eluted with elution buffer (2 M NaCl, 8 M urea, 50 mM Tris, pH 7.4, 500 mM imidazole, 0.01% sarcosyl, 5% trehalose). The elution was dialyzed against buffer (200 mM NaCl, 8 M urea, 50 mM NaAc, pH 5.2, 1 mM Dithiothreitol (DTT)) overnight. The P6.9 was then purified over 5-ml prepacked HiTrap SP FF column (SPA buffer: 200 mM NaCl, 8 M urea, 50 mM NaAc, pH 5.2, 1 mM DTT; SPB buffer: 2 M NaCl, 8 M urea, 50 mM NaAc, pH 5.2, 1 mM DTT). After purification, MBP-P6.9 fractions were dialyzed 4 times to remove urea and decrease NaCl concentration. The final buffer for P6.9 protein is 1 M NaCl, 50mM Tris (pH 7.5), 1 mM DTT, 5% trehalose, 0.01% sarcosyl, and 10% glycerol.

### Electrophoretic mobility shift assay

MBP-P6.9 (wild type and mutants) was incubated with 79-bp DNA (concentration of 500 nM) in the buffer of 50 mM Tris (pH 7.4), 100 mM NaCl, 1 mM EDTA, and 1 mM DTT at the molar ratio of 1:1, 2:1, 4:1, and 8:1 (MBP-P6.9:DNA). After incubation for 10 min on ice, the result was analyzed by 5% native PAGE and 4–15% sodium dodecyl sulfate–polyacrylamide gel electrophoresis (SDS–PAGE).

### Fluorescence polarization

To determine the binding affinity of P6.9 and DNA, varying amounts of P6.9 NA or NE (titration up to 7000 nM) were mixed with 20 nM Cy5-labeled 39-bp DNA, with the final buffer containing 50 mM HEPES (pH 7.5), 0.005% sarcosyl, 100 mM NaCl, 0.5 mM EDTA, and 1 mM DTT. The reaction was incubated in a 384-well microplate for 1 h at 4°C. Fluorescence polarization (FP) data (obtained on a CLARIOstar plus microplate reader from BMG Labtech) were analyzed by GraphPad Prism 9 (version 9.0.0). Three replicates were done. Absolute *K*_d_ values were obtained with labeled DNA.

#### Immunofluorescence microscopy

A total of 2 × 10^5^ Sf9 cells were seeded in a 35-mm glass-bottom culture dish (MatTek) and transfected with 1 μg vP6.9-HA and mutant recombinant virus bacmid DNA. At the desired h p.t., cells were washed three times with phosphate-buffered saline (PBS) and fixed with 4% paraformaldehyde in PBS for 10 min at RT. After washing three times with PBS, the fixed cells were permeabilized for 15 min at RT using 0.15% Triton X-100 in PBS containing 0.1% normal goat serum. Following three additional washes with PBS, the cells were blocked with 1% normal goat serum in PBS for 5 min, incubated with the primary antibody for 60 min, washed three times with blocking buffer, and then incubated with the secondary antibody for 60 min at RT. The secondary antibodies included goat anti-mouse IgG conjugated with Alexa Fluor 561 (Invitrogen Life Technologies) and goat anti-rabbit IgG conjugated with Alexa Fluor 647 (Invitrogen Life Technologies). Finally, the labeled cells were stained with 1 μg/ml Hoechst 33258 for 3 min at RT prior to analysis. All images were collected using a Leica SP8X confocal microscope according to the same parameters as those used for the mock-infected cells in each experiment.

#### Transcriptome sequencing and analysis

Transcriptome sequencing was performed by Beijing Novogene Technology Co., Ltd. The sample preparation process was as follows: Prepare six culture dishes, each containing 2 × 10^6^ cells. After cell attachment, infect the cells with recombinant virus bacmid DNA at a multiplicity of infection (MOI) of 10 TCID_50_/cell (concentrating to an equal volume by virus concentration reagent from Accurate Biotechnology), and include a group of uninfected cells as a negative control. After 1 h of virus infection, wash the cells once with a double-serum-free medium, and add 3 ml fresh insect cell culture medium. Incubate at 27°C for 24 h and then collect the cells. Wash the cells with PBS once, scrape them off, and centrifuge at 4°C and 4000 rpm for 10 min. Freeze the cell pellets in liquid nitrogen and store them temporarily at −80°C. RNA extraction and transcriptome sequencing were performed by the Guangzhou Technology Service Laboratory of Beijing Novogene Technology Co., Ltd. This experiment included five biological replicates, and due to the large number of samples, the replicates were sent for sequencing in five separate batches. Alignment of raw sequencing data and read quantification were performed using HISAT2 (version 9.0.0) and featureCounts (Subread version 2.0.4), respectively [26, 27]. The differential expression analysis was performed using DESeq2 (version 1.46.0) [28]. GO and KEGG annotations of the host proteome sequences were performed using the eggNOG-mapper (version 2.2.9) and KAAS web-based server, respectively [29, 30]. Subsequent GO enrichment and KEGG pathway enrichment analyses were conducted using non-parametric methods with the clusterProfiler package (version 4.12.6) [31].

#### Statistical methods

Comparative analysis of recombinant viral titers was performed using one-way analysis of variance (ANOVA) with multiple comparisons in GraphPad Prism 9 (version 9.0.0). The co-localization coefficients between P6.9 and host H4 were quantified using the Coloc2 plugin in ImageJ (Fiji distribution, version 1.53c), followed by statistical validation through one-way ANOVA with multiple comparisons in GraphPad Prism 9. Cell type classification following differential viral infections was conducted through morphological assessment, with subsequent one-way ANOVA and multiple comparisons applied to compare the proportional distributions across three defined cellular phenotypes.

## Results

### Hypo-phosphorylation of viral P6.9 N-terminus is crucial for the production of progeny viruses and its DNA-binding ability

**Figure 1.**
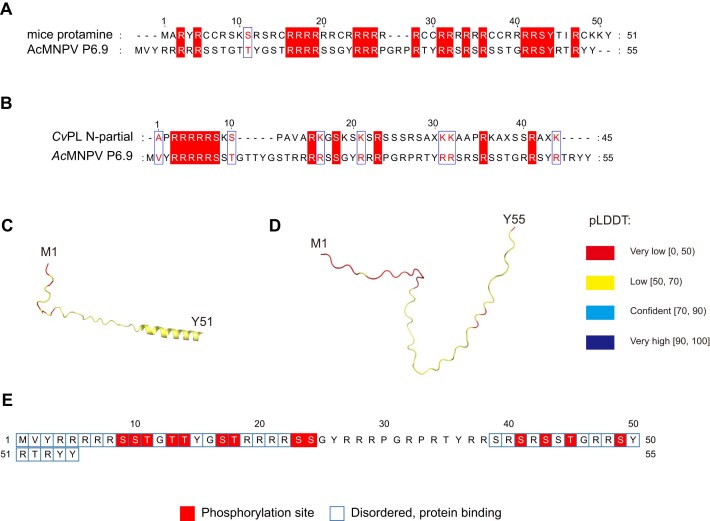
Sequence alignment and structural prediction of AcMNPV P6.9 and other SNBPs. (**A**) Sequence alignment of mice protamine [40] and AcMNPV P6.9 by ClustalW. (**B**) Sequence alignment of CvPL N-partial [33] and AcMNPV P6.9 by ClustalW. (**C**) The structure prediction of mice protamine by ColabFold. M1 represents the methionine at position 1, and Y51 represents the tyrosine at position 51. pLDDT indicates the confidence of each residue in the ColabFold predicted structure, ranging from 0 to 100. Residues with pLDDT ≥ 70 are universally correct structural predictions, whereas residues with pLDDT < 50 cannot be interpreted as structural information [38, 41]. (**D**) The structure prediction of AcMNPV P6.9 by ColabFold. M1 represents the methionine at position 1, and Y55 represents the tyrosine at position 55. (**E**) The IDRs prediction of AcMNPV P6.9 by DISOPRED3.

As a protamine-like protein, baculovirus P6.9 shares similar structure and function with protamine [10, 11]. We first tried to compare AcMNPV P6.9 with common protamine (including mammals and fish) or protamine-like protein by ClustalW [32]. The results (Fig. 1A and B) revealed a relatively conserved arginine content between mice protamine and AcMNPV P6.9, and similar basic amino acid composition between *Chaetopterus variopedatus* protamine-like protein (CvPL) and P6.9 [33]. It seems like the basic amino acid content of P6.9 is intermediate between typical/common protamine and protamine-like protein. Protamines usually do not have stable structure in diluted solution and physiological salt, and contain certain intrinsically disordered regions (IDRs) that are often involved in interactions with other proteins or nucleic acids [34–36]. In order to elucidate the regulatory mechanisms of P6.9, structural prediction of mice protamine and AcMNPV P6.9 using ColabFold (an online protein structure prediction tool based on MMseqs2 and AlphaFold2) was applied [37], and the results revealed overall low confidence scores for the amino acid residues (Fig. 1C and D). In the current research, long regions with pLDDT < 50 (per-residue local distance difference test score) should be interpreted as computationally predicted disordered domains rather than structural elements [38]. It supports the existence of substantial IDRs within the N-terminal region of P6.9. Therefore, we chose an online disorder prediction tool with good comprehensive performance—DISOPRED3 to predict the IDRs of P6.9 [39]. It was identified that the N-terminal residues 1–22 and the C-terminal residues 39–55 constitute the disordered regions. Notably, these regions also harbor the majority of P6.9’s serine/threonine phosphorylation sites (Fig. 1E).

**Figure 2.**
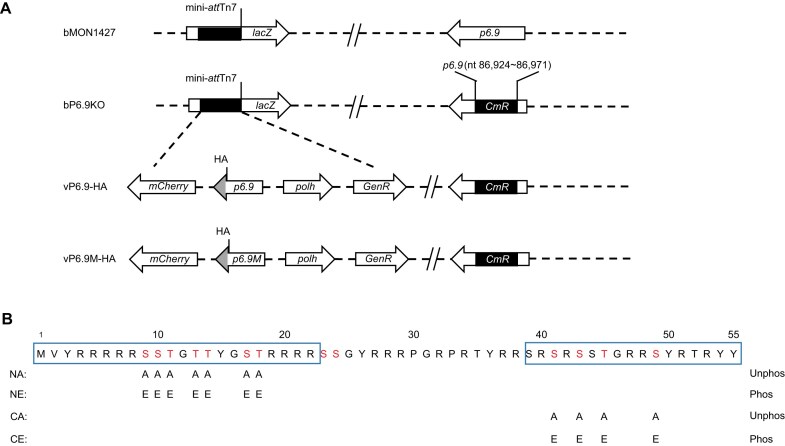
P6.9 mutant virus construction. (**A**) Schematic diagram of the AcMNPV recombinant viruses used in this study. The bP6.9KO (P6.9-deficient viral expression vector) was constructed by replacing a 48-bp fragment of p6.9 in bMON14272 (baculovirus shuttle vector) with a CmR gene. The vP6.9-HA (p6.9-rescued virus) was constructed by transposing the HA-tagged *p6.9* as well as the *polh* and *mcherry* genes into the bP6.9KO. Furthermore, vP6.9M-HA was constructed by mutating serine/threonine phosphorylation sites in P6.9 to alanine or glutamic acid. (**B**) Design of P6.9 mutation sites. NA/NE, substituting 7 serine or threonine sites at N-terminus with alanine/glutamic acid (7A/7E). CA/CE, substituting 4 serine or threonine sites at C-terminus with alanine/glutamic acid (4A/4E). Unphos, simulated hypo-phosphorylated forms of P6.9 by replacing serine/threonine with alanine. Phos, simulated hyper-phosphorylated forms of P6.9 by replacing serine/threonine with glutamic acid. Fluorescence microscopy of Sf9 cells transfected with the recombinant viruses.

Furthermore, given the existence of dozens of combinatorial phosphorylation isoforms of P6.9 [18], direct functional interrogation of these phospho-regulatory states is technically prohibitive. Based on the aforementioned results, we constructed P6.9 variants with simulated hyper-phosphorylation mutations (substituting serine/threonine with glutamic acid) and simulated hypo-phosphorylation mutations (substituting serine/threonine with alanine) to investigate whether phosphorylation regulation within these two regions perform distinct functional roles, at both the N-terminal region and the C-terminal region of P6.9, respectively (Fig. 2A and B). Based on the P6.9-deficient virus, we constructed rescued and mutant recombinant viruses. Additionally, an HA tag was fused to the C-terminus of both wild-type and mutant P6.9, while the *mCherry* fluorescent protein tag was transposed to monitor transfection/infection efficiency (Fig. 2A).

**Figure 3.**
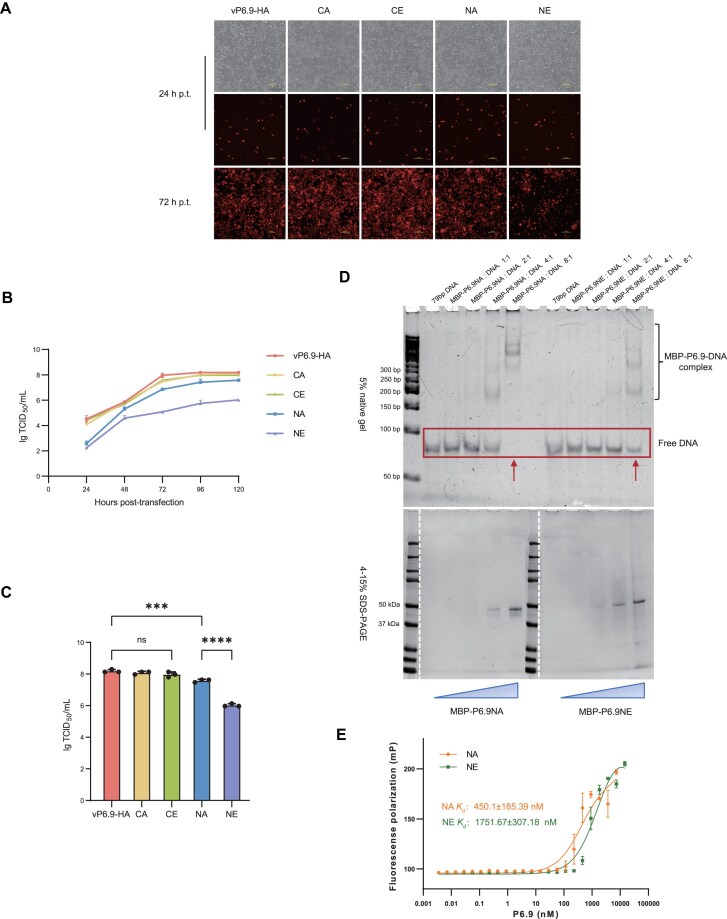
The viral proliferation determination and DNA binding ability of P6.9 and its mutants. Fluorescence microscopy of Sf9 cells transfected with the recombinant viruses (**A**), the growth curves of recombinant viruses (**B**), and the titers of their infectious progeny viruses at 120 h p.t. (**C**). The virus titers are displayed as averages in triplicate, with error bars indicating standard deviations (SDs). One-way ANOVA with multiple comparisons was used to compare the titer among recombinant viruses at 120 h p.t. Scale bar, 100 μm. ns, not significant. ***P < .001. ****P < .0001. (**D**) EMSA of MBP-tagged P6.9 mutants with 79-bp DNA. Since the C-term P-sites of P6.9 do not affect viral propagation, the N-term P-sites were primarily mutated to either A or E to mimic its hypo-phosphorylation (NA) or hyper-phosphorylation (NE). In the figure below, the blue triangles indicate a gradual increase in protein loading amounts, corresponding precisely to each well in the upper panel. (**E**) FP assay of P6.9 NA and NE (titration of 0–7000 nM) with 20 nM Cy5-labeled 39-bp DNA. *K*_d_, dissociation constant, it is a measure of the propensity of the DNA–protein complex to dissociate into their individual counterparts. *K*_d_ in nM: P6.9 NA 450.1 ± 185.39 (*R*^2^ = 0.9289); P6.9 NE 1751.67 ± 307.18 (*R*^2^ = 0.9620). Both the *K*_d_ and SDs were derived from three biological replicates.

To investigate how phosphorylation regulates the function of viral P6.9, it is essential to observe its influences on viral proliferation. One micro gram of recombinant viral ultra-pure bacmid DNA was transfected into 1 × 10^6^ Sf9 cells. At 24 h p.t., the proportion of cells expressing red fluorescent protein was roughly the same across all samples, indicating a consistent transfection efficiency. At 72 h p.t., the spread of the recombinant virus with C-terminal mutations (CA or CE) in P6.9 was unaffected in relation to the wild type, whereas the recombinant virus with N-terminal hyper-phosphorylated mutations (NE) exhibited the most notable reduction in spread, albeit not completely inhibited (Fig. 3A). The supernatant was collected up to 120 h p.t., the viral titers in the collected supernatants were measured using the TCID_50_ [23, 24], and the viral growth curves were plotted. Overall, the spread rate of the recombinant virus with N-terminal mutations was lower than that of the revertant virus (vP6.9-HA) and the C-terminal mutant viruses (Fig. 3B). One-way ANOVA and multiple comparisons of viral titers at 120 h p.t. revealed a significant difference between N-term hypo-phosphorylated mutation (NA) and N-term hyper-phosphorylated mutation (NE) groups. Similarly, a decrease in viral titers was observed between progeny virus of vP6.9-HA and NA. However, the viral titers of the C-terminal mutants’ progeny did not show a significant difference compared to vP6.9-HA (Fig. 3C).

The primary function of P6.9 is to package the viral genome into the viral capsid. Since the hyper-phosphorylation of P6.9 N-terminal region significantly affects viral propagation, it likely influences the binding of P6.9 to DNA. To verify this, MBP-tagged P6.9 mutants NA and NE were expressed, purified, and subjected to *in vitro* binding assays. The MBP tag could improve P6.9 solubility *in vitro*. Given that P6.9 theoretically exhibits no sequence specificity in packaging viral genomes, we selected random-sequence DNA to mix with P6.9 at different molar ratios. The electrophoretic mobility shifts of protein–DNA complexes were observed via electrophoretic mobility shift assay (EMSA) [42]. The native gel shown in the upper panel demonstrates the formation and electrophoretic mobility shifts of protein–DNA complexes, while the 4%–15% SDS–PAGE in the lower panel exhibits a consistent gradient in protein loading amounts (Fig. 3D). It suggested a better DNA binding of the hypo-phosphorylated (NA) P6.9 than the hyper-phosphorylated (NE) P6.9. Furthermore, to quantitatively compare the DNA-binding affinity differences between the NA and NE, we performed FP assay to monitor the binding of P6.9 to DNA and calculated the dissociation constant (*K*_d_) (Fig. 3E) [42]. As expected, the NA had a much higher binding affinity to free DNA compared to NE. This may be due to phosphorylation affecting the net charge of the N-terminal region. The results above indicated that the hyper-phosphorylation of the P6.9’s N-terminal region may primarily influence its affinity to DNA, and the transition to hypo-phosphorylation of the P6.9’s N-terminal region is crucial for virus proliferation.

### Hyper-phosphorylation of P6.9 N-terminus regulates its distribution pattern in the host nucleus

After viral infection, host and viral DNAs are distributed in different regions within the cell nucleus. Previous studies found that P6.9 accumulates significantly around the inner nuclear membrane and co-localizes with host chromatin [21]. The hyper-phosphorylation of the N-terminus can affect the binding of P6.9 to DNA, which likely influences its distribution within the nucleus. HA-tagged antibodies were used to indicate the localization of both wild-type and mutant P6.9, with histone H4 serving as a marker for host chromatin [21]. Additionally, Hoechst staining was used to visualize intracellular DNA for observing the VS region. Laser confocal microscope was employed to observe and analyze the effects of serine/threonine phosphorylation on the intranuclear distribution of P6.9.

**Figure 4.**
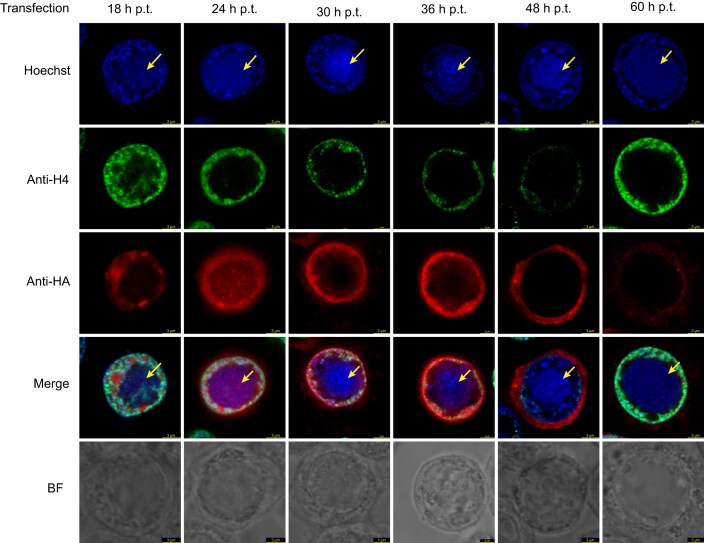
Localization of viral P6.9 during the infection cycle. Sf9 cells transfected with vP6.9-HA were immune-stained and scanned by laser confocal microscope at different time points from 18 to 60 h p.t. The VS region is indicated by arrows. BF, bright field. Scale bar, 3 μm.

After transfection, the distribution of wild-type P6.9 and host histone H4 in the cell nucleus was observed at different time points (Fig. 4). At 18 h p.t., a certain degree of marginalization of host chromatin was observed, and a small amount of P6.9 protein signal appeared around the inner nuclear membrane. At 24 h p.t., the host chromatin was further marginalized, while the P6.9 signal within the nucleus increased, gathering around the inner nuclear membrane. P6.9 signals were also observed in the VS region, showing some punctate aggregation. At 30 h p.t., the histone H4 signal began to weaken, which indicates marginalization of the host chromatin. The edges of the VS region became clearer, and the P6.9 signal within the nucleus further intensified, accumulating around the inner nuclear membrane, with only a small amount distributed in the VS region. At 36 h p.t., the H4 signal became faint and incomplete, but the distribution of P6.9 remained unchanged. At 48 h p.t., histone H4 signals were almost undetectable, while the P6.9 signal had moved from the nucleus to the cytoplasm. By 60 h p.t., the P6.9 signal became attenuated again, whereas H4 signals re-emerged with distinct localization at the inner nuclear membrane periphery. This reciprocal pattern suggests the initiation of a new cycle of viral infection. Therefore, to achieve a strong P6.9 signal and clear marginalization of host chromatin regions and VS regions, the optimal observation time for this study was 30 h p.t.

**Figure 5.**
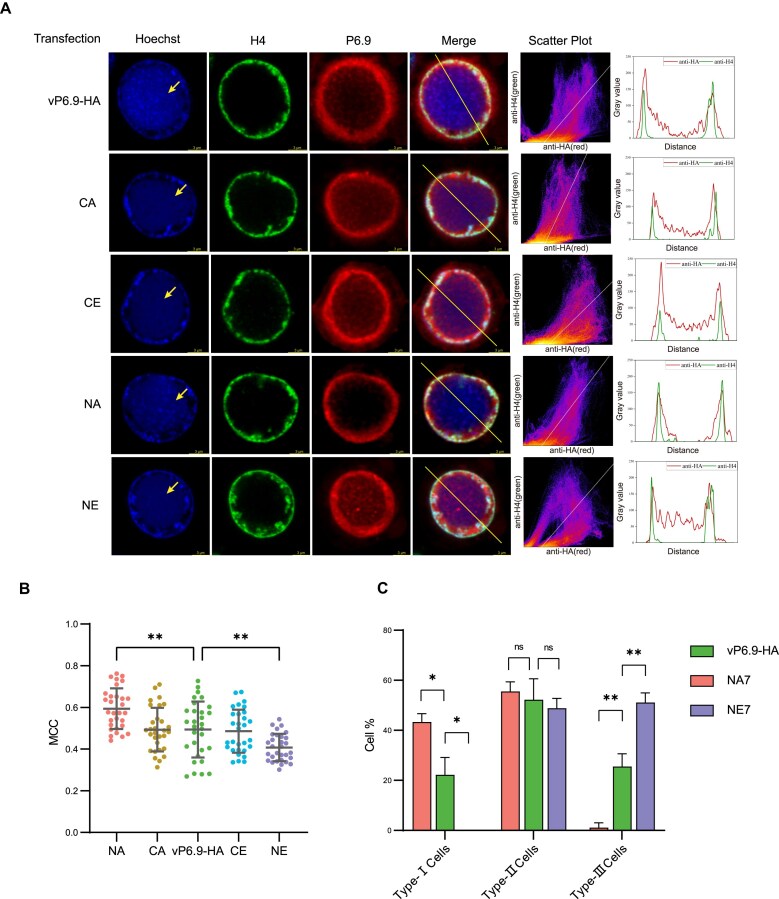
The distribution of P6.9 mutants in the nucleus under laser confocal microscopy. (**A**) One microgram bacmid DNA of the recombinant viruses was transfected into 6 × 10^5^ Sf9 cells. The cells were immune-stained and scanned by laser confocal microscope at 30 h p.t. The fifth column represents frequency scatter plots of the intensity registered in the red and green channels. The last column shows signal intensity profiles for green and red channels along the yellow line in the picture that is depicted in the merge channel. The VS region is indicated by arrows. Scale bar, 3 μm. (**B**) During laser confocal microscope observation, 30 cells were photographed per sample. The Manders co-localization coefficients (MCCs) are displayed as the dots for 30 cells and their mean value, with error bars indicating SDs. One-way ANOVA with multiple comparisons was used to compare the MCC among the p6.9-mutated viruses and the p6.9-rescued virus. The results were validated by three independent biological replicates. (**C**) The number of cells of each type was counted and calculated as a percentage of the total 30 cells for each experiment. The ratios of different cell types are displayed as averages in triplicate. One-way ANOVA with multiple comparisons was used to compare the cell ratio among the p6.9-mutated viruses and the p6.9-rescued virus. ns, no significance. *P < .05. and **P < .01.

A total of 1 μg of each recombinant virus bacmid DNA was transfected into 6 × 10^5^ Sf9 cells, and the cells were fixed and permeabilized for immunostaining at 30 h p.t. Three biological replicates were performed, and 30 cells from each sample were imaged per replicate. The scatter plot and fluorescence intensity distribution (Fig. 5A) showed that similar to P6.9-HA, the C-terminal mutant P6.9 is primarily distributed around the inner nuclear membrane, while some co-localized with host H4 and a small amount localized in the VS. Intriguingly, the distribution of NA in the VS region is significantly reduced, while NE shows a notable increase in the VS region. This suggests that the phosphorylation state of P6.9 N-terminal region affects its distribution within the nucleus.

Furthermore, to quantitatively analyze the co-localization of host H4 and viral P6.9 in each of the 30 cells imaged and to examine the binding of P6.9 to host DNA, MCCs were applied to describe the co-localization of P6.9 with the host chromatin [43]. These data represent the proportion of P6.9 signals that co-localize with H4 relative to the total P6.9 signals in the nucleus. Compared to the wild-type P6.9, the phosphorylation state of the C-terminal region does not affect the co-localization of P6.9 with host chromatin (Fig. 5B). Strikingly, hypo-phosphorylation of the N-terminal region (NA) significantly increased the co-localization of P6.9 with host chromatin, while hyper-phosphorylation of the N-terminus (NE) had the opposite effect. Therefore, the phosphorylation of the P6.9’s N-terminal region can influence its relationship with host chromatin.

The above results do not allow for quantitative analysis of P6.9 distribution in the VS region in virus-transfected/infected cells. Since it is difficult to specifically label the DNA in the nuclear VS region, we categorized the cells into three types according to the distribution of P6.9 in the host cell nucleus: Type I cells, where P6.9 is only distributed around the inner nuclear membrane; Type II cells, where P6.9 is predominantly distributed around the inner nuclear membrane with a small amount in the VS region; and Type III cells, where P6.9 is somewhat concentrated around the inner nuclear membrane but is largely distributed in the VS region in a diffuse pattern. The proportions of each cell type in different samples across three experiments were statistically analyzed, but no significant difference in the proportions of the three cell types was observed after C-terminal mutations of P6.9 compared to the wild type (data not shown). Interestingly, the hypo-phosphorylated N-terminal region of P6.9 (NA) caused the proportion of Type I cells to significantly increase, while the proportion of Type III cells significantly decreased. In contrast, the hyper-phosphorylation N-terminal region of P6.9 (NE) caused the proportion of Type I cells to significantly decrease, while the proportion of Type III cells significantly increased (Fig. 5C). Altogether, hypo-phosphorylated P6.9 at the N-terminal region accumulates more around the inner nuclear membrane and co-localizes with host chromatin, whereas hyper-phosphorylated P6.9 at the N-terminal region moves away from the marginal host chromatin region and enters the VS region, where P6.9 performs its functions.

### Phosphorylation of viral P6.9 N-terminus participates in gene transcription regulation

**Figure 6.**
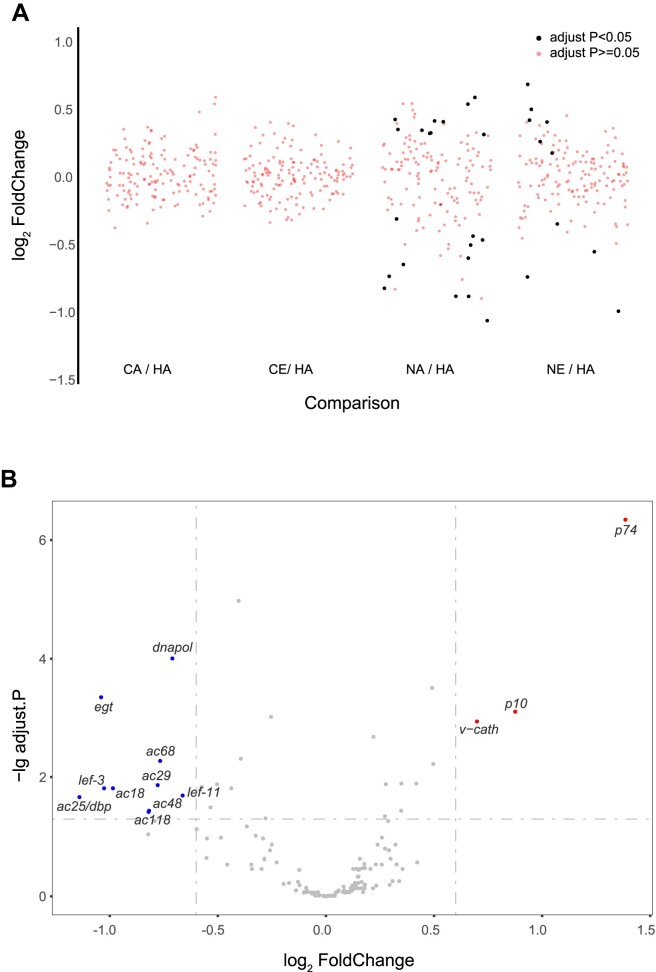
RNA sequencing and differential expression analysis of P6.9 and its mutants on viral genes. (**A**) Volcano plot showing multiple comparisons on *x*-axis. (**B**) Volcano plot of the DEGs between the recombinant virus NA and NE. DEGs were determined using a threshold of adjust. *P* ≤ .05 and |log_2_ FoldChange| ≥ 0.6.

Overexpression of P6.9 can increase the transcription levels of certain viral genes in the late stages of infection (12–24 h p.t.) [44]. Additionally, AcMNPV protein kinase 1 (PK1) can specifically phosphorylate certain serine/threonine sites in P6.9, and mutations at these sites affect the transcription of very late viral genes [18]. Therefore, as a DNA-binding protein, the phosphorylation of P6.9 may contribute to gene silencing or transcriptional regulation. To confirm this, the Sf9 cells were infected with different recombinant viruses at MOI of 10 TCID_50_/cell and collected at 24 h p.i. RNA sequencing and differential expression analysis were then conducted to investigate the effect of P6.9 phosphorylation on gene expression. To compare the viral gene transcriptional level, differential expression analysis of mutant viruses was conducted by using vP6.9-HA as a control, and only NA and NE showed significantly differentially expressed genes (DEGs) (Fig. 6A). No significant DEGs are detected between the CA and CE (data not shown). Intriguingly, NA detected some DEGs when compared to NE (Fig. 6B), indicating that the hypo-phosphorylation of the P6.9 N-terminus is involved in the transcriptional regulation of viral genes. Most DEGs in NA were downregulated, and some of the downregulated genes were associated with viral DNA replication (e.g. ac25), suggesting that hypo-phosphorylation at the P6.9 N-terminal region can inhibit the expression of viral DNA replication-related proteins. Additionally, a decrease in the transcriptional levels of some viral late expression factors, such as lef-3 and lef-11, was observed. Therefore, phosphorylation dynamic regulation of the P6.9 N-terminal region likely influences viral DNA replication and the transcriptional expression of viral late genes.

**Figure 7.**
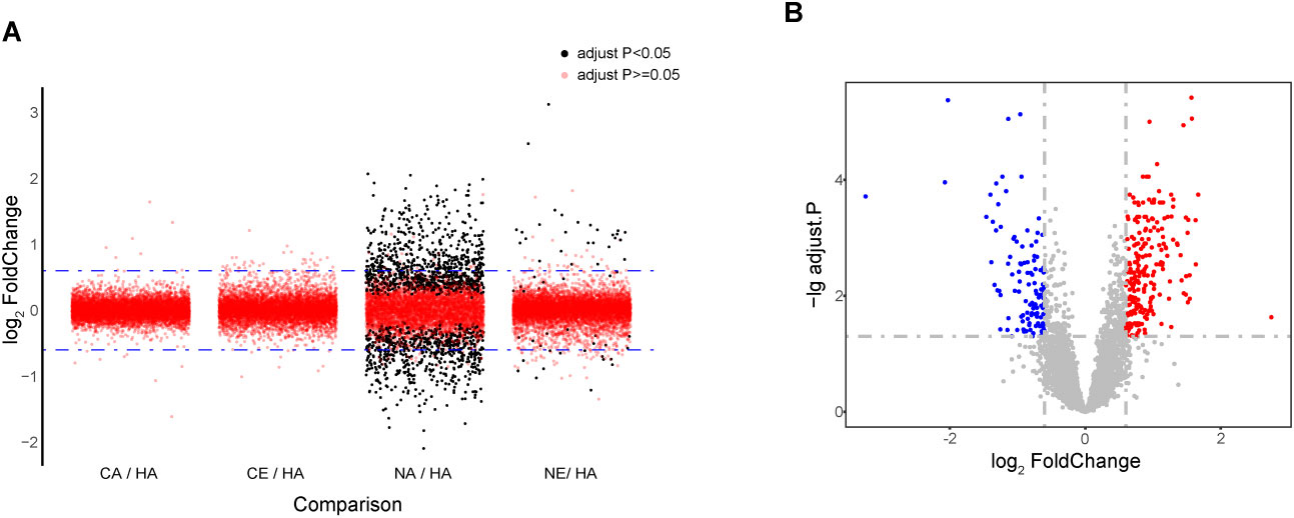
RNA sequencing and differential expression analysis of P6.9 and its mutants on host genes. (**A**) Volcano plot showing multiple comparisons on the *x*-axis. The lines represent |log_2_ FoldChange| = 0.6. (**B**) Volcano plot of the DEGs between the infected cells with recombinant viruses NA and NE. The lines represent a threshold of adjust. *P* ≤ .05 and |log_2_ FoldChange| ≥ 0.6.

Although the phenomenon of host chromatin marginalization during baculovirus infection is unrelated to P6.9 [21], a significant amount of P6.9 accumulates around the inner nuclear membrane throughout the infection process (Fig. 4). Research has suggested that the overall transcriptional level of host genes is downregulated after infection [45, 46]. The process may be related to P6.9 accumulation around the inner nuclear membrane. For the transcriptome analysis of the host gene, using host cells infected with the vP6.9-HA as a control, gene expression in cells infected with mutant viruses was analyzed (Fig. 7A). Only cells infected with the recombinant virus carrying the mutation at the P6.9 N-terminus showed significant DEGs, and significant upregulation or downregulation of gene expression was detected between the host cells infected with NA and NE, respectively (Fig. 7B). Not surprisingly, there are no significant DEGs between the host cells infected with CA and CE (data not shown), which strongly indicates that the phosphorylation sites in the P6.9 N-terminal region possess a transcriptional regulatory role. Enrichment analysis was then performed on all significant DEGs between the two groups (NA and NE) to explore the potential biological processes involved.

**Figure 8.**
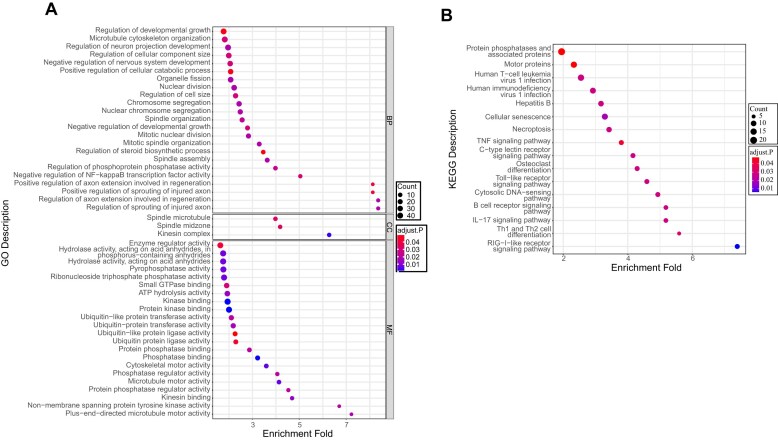
Enrichment analysis of all significant DEGs between the NA and NE. (**A**) Bubble plot of GO enrichment analysis of DEGs in host cells after infection with P6.9 mutants in the N-terminal region. (**B**) Bubble plot of KEGG enrichment analysis of DEGs in host cells after infection with P6.9 mutants in the N-terminal region.

GO enrichment analysis revealed (Fig. 8A) that the DEGs were primarily enriched in two major categories: molecular function and biological process. Among the significantly enriched GO terms, activities related to protein kinases and phosphatases showed high significance, all of which are associated with protein phosphorylation. This suggests that the virus may regulate host protein kinases and phosphatases through the phosphorylation of P6.9 to further affect the phosphorylation of other viral or host proteins. Additionally, a considerable number of DEGs were enriched in functions related to ubiquitin ligases. As a PTM with complex mechanisms and diverse effects, ubiquitination regulates processes such as transcriptional regulation, DNA damage repair, cell cycle, and apoptosis by affecting protein stability, localization, activity, and interactions [47–52]. KEGG enrichment analysis revealed that the DEGs were mainly enriched in protein phosphatases and associated proteins, with several other important signaling pathways detected as well (Fig. 8B). For example, cellular senescence is typically an adaptive response induced by physiological or pathological stress factors, leading to cell cycle arrest [19]. Activation of the RIG-I-like receptor signaling pathway promotes cytokine production, thereby exerting antiviral effects [53]. These results suggest that phosphorylation regulation of the P6.9 N-terminal region is highly likely involved in regulating protein PTM-related and immune response-related pathways.

## Discussion

**Figure 9.**
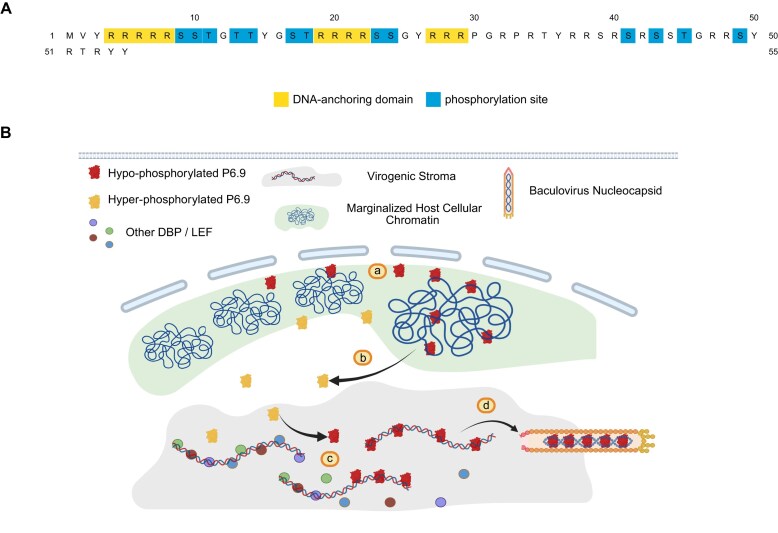
Proposed model of P6.9 phosphorylation/dephosphorylation dynamics. (**A**) Phosphorylation sites and DNA-anchoring domains of P6.9 [18, 21]. (**B**) A potential model in which P6.9 participates in the virus genome packaging and regulation of gene expression by reversible phosphorylation in its N-terminus: (a) hypo-phosphorylated P6.9 enters the host cell nucleus and binds host chromatin; (b) P6.9 translocates into VS after phosphorylating within its N-terminal region; (c) hyper-phosphorylated P6.9 undergoes dephosphorylation and binds the viral genome so as to regulate the expression of viral genes; and (d) totally dephosphorylated P6.9 packs the viral genome into the capsid.

In virus-infected cells, P6.9 is phosphorylated shortly after synthesis [54]. As infection progresses, the amount of phosphorylated P6.9 gradually increases and various phosphorylated forms emerge [21]. Our research found that the binding capability of the hyper-phosphorylated P6.9 N-terminal region to DNA significantly decreases compared to hypo-phosphorylation (Fig. 3E). P6.9 contains three polyarginine segments in the N-terminal region, each comprising more than three consecutive arginine residues that are inferred to function as DNA anchoring domains (Fig. 9A) [21]. Studies have shown that inserting negatively charged amino acids between polyarginine segments reduces its affinity for DNA, thereby affecting its ability to compact DNA [55, 56]. Molecular dynamics simulation reveals that the presence of phosphorylated serine between polyarginine segments could reduce the binding efficiency of these segments to DNA and cause the segments to form a more compact conformation [57]. This process is probably due to the phosphate groups of serine competing with DNA for arginine residues. Consequently, this alteration makes the phosphorylated peptide segments more prone to invading chromatin [57]. Research work has demonstrated that the basic group of arginine and lysine plays an essential role in the stability of interactions between nuclear basic proteins and DNA [58, 59]. Therefore, the reversible phosphorylation of the N-terminal region could affect the local charge of P6.9 and is key to the viruses to regulate their DNA binding affinity.

In metabolic regulation, phosphorylation is arguably the most commonly activated post-translational modification to regulate the activity or subcellular localization of target proteins [17, 60], such as the phosphorylation of pyruvate dehydrogenase E1 subunit alpha (PDHE1α) and the regulation of its localization in the cytoplasm and mitochondria [61]. A previous study observed that protamine also localizes around the inner nuclear membrane during spermatogenesis, a process essential for protamine phosphorylation [62]. Shortly after protamine synthesis, the serine and threonine residues within it are phosphorylated, which assists in replacing core histones and other chromatin proteins [63]. During the subsequent sperm maturation process, protamine is then dephosphorylated, enabling its function in compacting the genome [64, 65]. In this study, hyper-phosphorylation at the P6.9 N-terminal region makes it translocate from the marginal host chromatin to the VS (Fig. 5). Notably, only dephosphorylated P6.9 is present in mature virions [21], which is why the titers of NE progeny virus decreased the most (Fig. 3C). Therefore, a process similar to spermatogenesis may occur during baculovirus replication. After synthesis, hypo-phosphorylated P6.9 accumulates at the periphery of the inner nuclear membrane, a process that may be intimately linked to subsequent phosphorylation events. Corresponding to it, the hyper-phosphorylation of the P6.9 N-terminal region may be necessary for its translocation to the VS. Current findings established that the viral PK1 and protein kinase-interacting protein (PKIP) are critically involved in phosphorylating P6.9, with emerging evidence suggesting potential recruitment of additional viral and host factors in this process [18, 66]. Later on, phosphorylated P6.9 undergoes dephosphorylation to bind and compact the viral genome, thus completing viral proliferation. This also results in the unique phosphorylation homeostasis of P6.9 during viral infection. The viral protein 38K is exclusively known to mediate dephosphorylation [67]. Further details regarding this regulatory mechanism remain to be elucidated. Unexpectedly, for the phosphorylation sites at the C-terminus, neither hyper-phosphorylation nor hypo-phosphorylation affects the distribution of P6.9, gene expression, or the production of progeny viruses. Since the C-terminal region of P6.9 contains sequence elements essential for the formation of viral particles, these elements may interact with other nucleocapsid proteins during virus assembly rather than engage in DNA binding directly [14, 67].

Previous studies have demonstrated that during the late phase of baculovirus infection, the viral PK1 mediates multisite phosphorylation of P6.9. Disruption of phosphorylation at these critical residues markedly compromises the expression of viral very late genes [18]. It is noteworthy that the hypo-phosphorylated N-terminal region of P6.9 suppresses the transcription of certain viral genes at 24 h p.i., which are largely associated with viral DNA replication and late gene expression. (Fig. 6B). Among these genes, ac25, which encodes a DNA binding protein known as a single-stranded DNA binding protein (SSB) and is part of the VS, exhibited the highest differential expression. AC25 (usually called DBP) can unwind and anneal DNA, but is not essential for DNA replication [68]. Moreover, DBP could stabilize the viral genome before it is packaged into virions by P6.9 [20]. Additionally, the transcription levels of some late viral transcription factors, such as lef-3 and lef-11, were observed to be downregulated. LEF-3 encodes an SSB and is essential for viral DNA replication and VS formation [69, 70], which has a competition with DBP for binding sites on ssDNA templates [68]. LEF-11 promotes the transcription of late viral genes but is not necessary for immediate DNA replication [71]. Research on *Bombyx mori* nucleopolyhedrovirus has shown that LEF-11 co-localizes with IE1 at viral DNA replication sites in the cell nucleus and interacts with LEF-3 [72]. On top of that, the phosphorylation of P6.9 is required for the release of the viral genome and the binding of transcription factors during the early stages of infection [73–75]. These suggest that hypo-phosphorylated P6.9 may compete with other DNA-binding proteins or transcriptional regulators for binding sites, thereby perturbing downstream late gene expression (like p10). Altogether, the hypo-phosphorylation of P6.9 N-terminal region potentially inhibit viral DNA replication and regulates viral gene expression by suppressing the expression and binding of other DNA-binding proteins or transcription factors. These may lead to decreased titers of the progeny viruses of NA (Fig. 3C). We also found that N-terminal phosphorylation of P6.9 can influence the expression of host pathways related to PTMs, suggesting a close association between viral protein modification and host functional gene expression.

As one kind of SNBPs, protamine is widely present in the sperm of both plants and animals and is responsible for compacting and packaging DNA during sperm maturation [4, 5]. Mammalian protamines typically contain 50%–60% arginine without other basic amino acids, while fish protamines usually contain 65%–70% arginine [5]. On the other hand, some shellfish in the ocean contain a variety of PL proteins with a rich arginine + lysine content (Arg + Lys = 35–50 mol%) [34, 76]. P6.9, as the only nuclear basic protein of baculovirus, contains only 40% arginine and lacks other basic amino acids. Nevertheless, P6.9 contains a significant amount of serine + threonine (31%), which is substantially higher than in other species. Bovine protamine contains only 9.8% serine/threonine, salmon protamine has 12.9%, and mussel PL proteins contain ∼14%. Since the phosphorylation of P6.9’s serine/threonine could significantly affect its binding ability to DNA and cellular distribution, P6.9 appears to be a specialized protamine/PL protein. There may be a “histone—protamine-like protein—protamine” evolutionary pattern among SNBPs [76, 77], and the research on P6.9 contributes to our understanding of the functional regulation and evolutionary relationships among SNBPs.

Here we propose a model of viral P6.9 functional dynamics regulated by phosphorylation/dephosphorylation (Fig. 9B): (a) P6.9 carries substantial positive charges due to its high arginine content. After synthesis in the cytoplasm, P6.9 needs to be transported to the VS within the nucleus to package the viral genome. With a strong binding affinity to DNA, it would initially bind to the host chromatin and is probably related to the overall transcriptional level regulation of host genes. (b) P6.9 undergoes phosphorylation shortly after its synthesis. The N-terminal region of P6.9 has three DNA-anchoring domains, and the phosphorylation within these regions significantly affects the local charges, thereby reducing its DNA binding affinity. This allows P6.9 to detach from the marginalized host chromatin regions and enter the VS within the nucleus. Although P6.9 exhibits heterogeneous phosphorylation states during baculovirus infection, we posit that its primary DNA-binding regulatory sites reside in the N-terminal region. (c) The hyper-phosphorylated P6.9, due to its reduced affinity for the DNA, allows space for other DNA-binding proteins or transcriptional regulators to regulate viral genome replication and the expression of late genes. By cycling between phosphorylated states, VS-localized P6.9 may act as a molecular switch coordinating viral DNA packaging efficiency with late gene expression timing. (d) Given that viral nucleocapsids exclusively package non-phosphorylated P6.9, this protein must ultimately undergo complete dephosphorylation, then binds/compacts the viral genomic DNA, and completes the encapsidation process for the infection cycle [20].

*Limitations of this study*: Experimental evidence has demonstrated that GFP-tagged P6.9 fails to rescue P6.9-deficient viruses [44], likely due to excessive molecular weight discrepancy introduced by the fusion tag, which simultaneously restricts our ability to directly visualize the temporalspatial dynamics of distinct phosphorylation states of P6.9 within the nucleus. In the DNA binding assay of P6.9, we fused an MBP tag in our *in vitro* studies to assist solubilization due to the low solubility of P6.9, which may introduce interference in subsequent investigations into how phosphorylation modulates the structural dynamics of P6.9. Moreover, the time-dependent expression of baculovirus gene may introduce bias in bulk RNA-seq results due to heterogeneity in infection progression. Incomplete genome annotation of insect cells coupled with limitations of current sequencing platforms poses technical challenges for implementing single-cell transcriptome sequencing and related bioinformatic analyses. In addition to the multiple phosphorylation sites identified in P6.9, several methylation sites have also been discovered [18]. However, the interplay between these sites and their regulatory roles remains to be elucidated.

## Data Availability

All data relevant to the study are included in the article. The RNA sequencing data are uploaded to NCBI-SRA, and the BioProject number is PRJNA1222785.
